# Prospective, randomised clinical trial on the necessity of using a silicone intubarium in the context of endonasal-endoscopic dacryocystorhinostomy (EN-DCR) in patients with postsaccal lacrimal duct stenosis

**DOI:** 10.1007/s10792-024-03205-7

**Published:** 2024-06-28

**Authors:** Lia Bahner, Veit Zebralla, Andreas Dietz, Mathias Otto, Markus Pirlich

**Affiliations:** University Hospital Leipzig, Leipzig, Germany

**Keywords:** EN-DCR, SST, PANDO, Epiphora, Irrigation, Success rate

## Abstract

**Background:**

This prospective clinical study evaluates the effect of a silicone stent tube (SST) on the success rate of endonasal-endoscopic dacryocystorhinostomy (EN-DCR) to treat primary acquired nasolacrimal duct obstruction.

**Methods:**

Patients were randomly assigned to receive EN-DCR with or without SST intubation over a period of 3 months. The surgery was performed using standardized techniques. Patients were assessed at three different timepoints: one day, 12 weeks and 24 weeks after the surgery. The results were compared in order to evaluate statistical differences. Surgical success was determined by means of positive irrigation procedures, as well as by the improvement of symptoms and a high level of patient satisfaction.

**Results:**

A total of 56 randomized cases completed 24 weeks of follow up. 1 Patient dropped out due to malignant genesis of the nasolacrimal duct obstruction. After 24 weeks of follow up no statistically significant differences in levels of epiphora (*p* > .10) or patency (*p* > .16) were revealed. Comparisons regarding changes in time did not show levels of significance (*p* > .28).

**Conclusions:**

This study could not confirm a statistically significant benefit or disadvantage for SST Insertion in EN-DCR.

## Introduction

External and endonasal endoscopic dacryocystorhinostomy are standard techniques to treat obstruction of the nasolacrimal duct. An external approach was first described by Addeo Toti in 1904 which has since then been modified several times [1]. The first endonasal procedure to treat this pathology was conceptualized by Caldwell in 1893 and modified by John West in 1914. [2] Endonasal endoscopic Dacryocystorhinostomie (EN-DCR) was introduced in patients by McDonogh and Meiring in 1989 [3] and has shown additional advantages like prevention of an external scar, reduced post-surgical morbidity, reduced operative time, minimal bleeding, and early recovery. [4, 5] Since then, endoscopic DCR has become increasingly popular, especially due to technical advances in endoscopic equipment and improvements of surgical instruments in rhinologic surgery.

The procedure of Dacryocystorhinostomy (DCR) is based on the formation of a fistula on the lateral wall of the nose between the nasal cavity and the lacrimal sac in order to relieve an obstruction of the lacrimal drainage. EN-DCR provides similar results compared to external approaches, with a success rate of 83–94% and low risk of complications. [6, 7] Nevertheless EN-DCR can lead to the formation of granulation tissue, fibrosis or local synechia between the ostium and nasal septum, all of which can ultimately be the cause of failure of the procedure. [8] In order to provide the best possible outcome, different techniques and devices have been developed over the years. Varying studies have shown higher success rates for endoscopic DCR, when comparing external and endoscopic approaches in DCR surgeries. [4, 6] Indeed, success rates seem to be on the rise over the last decades due to deeper anatomical understanding, better diagnostic work-ups, higher quality instruments and imaging systems as well as the improved understanding of nasolacrimal healing [9].

Numerous adjuvant treatments including antifibrotic agents like 5-Fluorouracil, Mytomycin C, 5-Fluorouracil and Steroids, varying absorbent packaging materials like Gelfoam [Pharmacia & Upjohn Co, New York, N.Y.] and Merogel [Medtronic Xomed, Jacksonville, Fla.] as well as different silicone stent tubes (SST) have been used to provide a better outcome by preventing the size of the internal ostium from reducing. Especially the use of SSTs is controversially discussed. Stents of different materials have been tested for their ability to provide patency of the ostium. The very earliest Stent by Graue consisted of a silver wire, inserted through the lower punctum into the nose. [10] Over the years different organic (hair, catgut), metal (silver wire, Veirs’ rod) and synthetic (nylon, polyethylene, supramid, silicone and Teflon) materials have been tested to improve patency. So far, silicone can be considered the stent material offering the most advantages in terms of flexibility, durability, inertness, and tolerability [11].

Some studies considered SSTs to be the cause of tissue granulation, inflammation, and fibrosis, leading to restenosis which makes it necessary to perform revision surgery. Moreover, SSTs may cause discomfort for the patient, as they are a foreign body, even causing corneal erosion. Likewise, displacement is an added risk. Further treatment is necessary for the patient, as the stent has to be removed eventually after the procedure [12–15, 36].

Given a lack of prospective randomized trials [16] the purpose of this randomized clinical study was to evaluate the surgical outcome of endonasal-endoscopic DCR with and without the insertion of a SST and to determine whether a SST offers an advantage in endoscopic DCR.

## Material and methods

Patients with postsaccal stenosis of the nasolacrimal duct, who were referred to the tear outpatient clinic of the ophthalmology department of the University Hospital were recruited for the purpose of this prospective, randomized clinical trial. The study was conducted in accordance with the Declaration of Helsinki and approved by the Clinical Research Ethics Committee DE/EKSN40. Detailed information was provided to patients regarding surgery and randomization, and informed consent was obtained from participants. Both eyes of the eligible patient were permissible for testing. A slit-lamp examination of the tear film, eyelid, puncta, and anterior segment of the eye was performed to rule out reflexive tearing, palpebral fissures, and punctual constriction. The presence of lacrimal duct obstruction was determined by lacrimal duct probing and irrigation. The diagnosis of postsaccal obstruction was verified by an ophthalmologist: the occurrence of reflux during the irrigation of the efferent lacrimal duct system indicated the necessity for surgical intervention. Nasal examination including anterior rhinoscopy and nasal endoscopy was performed by an otolaryngologist to rule out significant nasal pathology.

Inclusion criteria for this study were (1) age > 18 years (2) diagnosis of postsaccal nasolacrimal duct stenosis (3) informed consent for the study and randomization. Exclusion criteria were (1) traumatic or malignant genesis of lacrimal duct obstruction and (2) women during pregnancy or lactation. Randomization was carried out one day before surgery by an independent institute for statistics and clinical studies.

Surgery was performed under general endotracheal tube anesthesia as an inpatient procedure. In a first step of the EN-DCR, a caudally stemmed mucoperiosteal flap was created on the lateral nasal wall anterior to the insertion of the middle turbinate using a sickle knife and a Freer elevator. After exposing the lacrimal bone to the maxillary line, bone material was removed using a Kerrison punch. The lacrimal sac was exposed with a diameter of at least 1 cm^2^. Using a lacrimal sac probe inserted into the inferior or superior punctum, the lacrimal sac was striated endonasally (“tenting”). The lacrimal sac was then incised with a sickle knife and the medial wall of the lacrimal sac was removed by means of sharp cutting instruments. After checking for adequate drainage of irrigation fluid into the nose, one sub-group received SSTs, which were inserted through the superior and inferior puncta and secured with sutures in the nasal cavity. The other sub-group remained without a SST after the irrigation. All study participants received nasal packages, which were removed during a routine clinical examination by an otolaryngologist on the first postoperative day. Patients underwent additional routine clinical examinations by an ophthalmologist and received irrigation of the lacrimal system on the first postoperative day. Antibiotic eye drops containing ofloxacin were prescribed twice per day over a period of 7 days. In addition, the use of topical nasal steroids and ointment containing panthenol were recommended over a period of 3 months. Regular check-ups with an otolaryngologist and ophthalmologist were recommended in order to remove endonasal crusting. After 12 and 24 weeks a nasal examination was performed in our department and irrigation of the nasolacrimal duct, as well as an eye examination was performed by an ophthalmologist in our hospital. After 12 weeks the SST was removed, if present. The functional and anatomical results were documented. Maximum ostium shrinkage occurs in the first month after which, average size of ostium is mostly stable with little change [17].

Both objective and subjective factors were collected in this study. For the subjective assessments patients were asked to rate the severity of following symptoms on a scale from zero (no symptoms) to five (a lot of symptoms): epiphora, recurrent inflammation of the eye, encrustations of the nose and olfactory reduction. This was done at four specific timepoints: prior to surgery, one day, three months and six months after surgery. During the follow-up sessions patients were additionally asked to grade their overall satisfaction with the procedure on a scale from zero (very unsatisfied) to five (very satisfied). Further symptoms, such as conjunctivitis, dacryocystitis, nasal airway obstruction among others were recorded. The objective assessment examined the patency of the nasolacrimal duct system by irrigation, which was performed by an ophthalmologist at each of the patients’ visit (prior to the operation, as well as one day, three months and six months after the surgery).

**Fig. 1:**
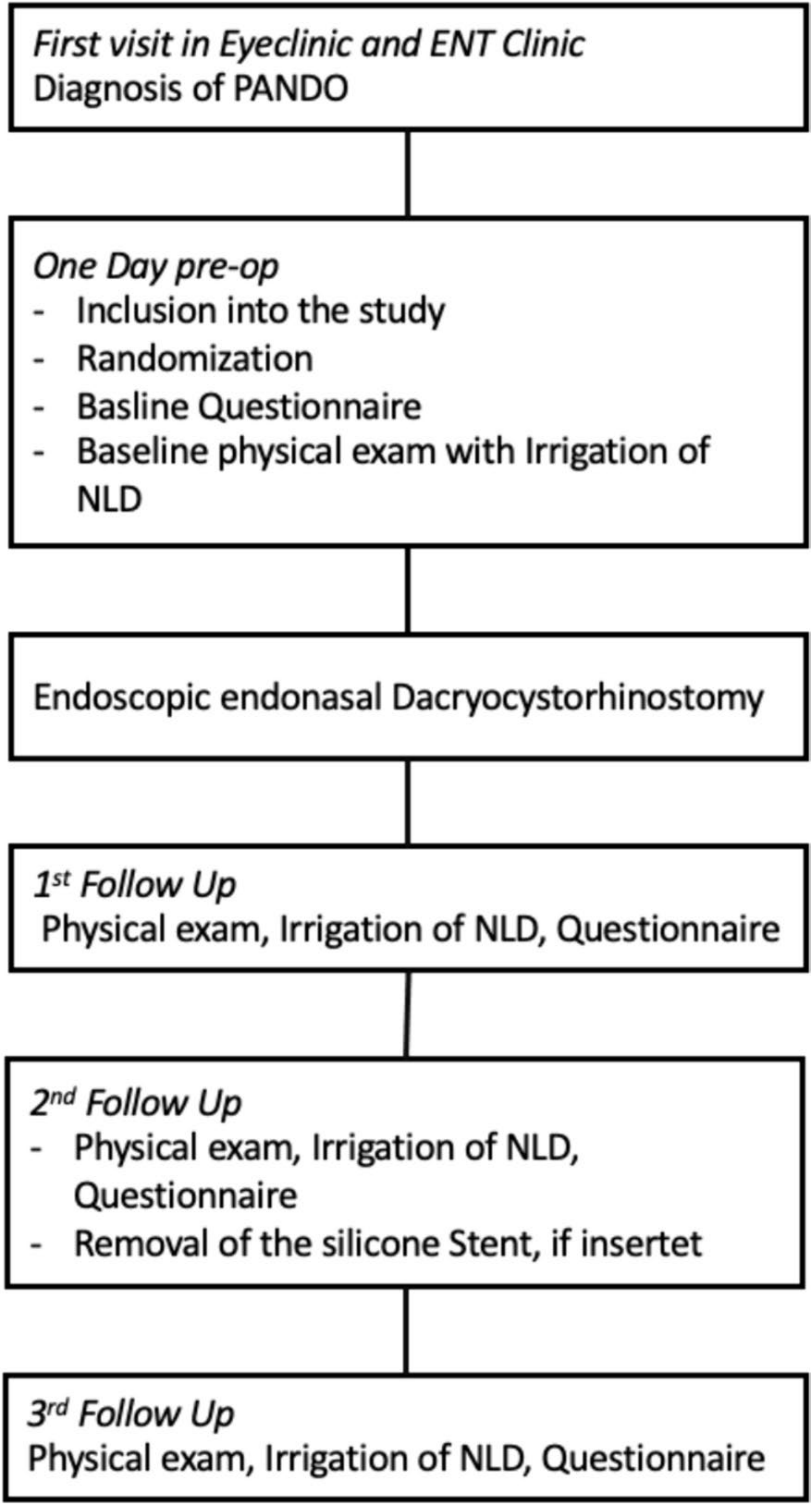
Description of the clinical prospective study design

Surgical success was subsequently more precisely differentiated into anatomical and functional success. Anatomical success was defined as postoperative patency of the NLD, tested by irrigation. Functional success was defined by a relief of symptoms (epiphora and recurrent inflammation) and patient satisfaction with the procedure of at least four or five (on a scale from zero to five) (Fig. 1).

Data preparation and analysis were performed using SPSS (Version 28) and Excel. Descriptives were given as proportions (categorical variables) and as mean values and standard deviations (*SD*; continuous variables). Linear- regression and Chi-square tests were carried out. For all analyses respective *p*-values were reported. The significance level *α* was set to alpha = 5%.

## Results

Overall, 57 patients, 11 male (19.3%) and 46 females (80.7%) were included in the study. The mean age of patients was 68.65 (SD = 17.65). In total 62 procedures were carried out, 32 on the left eye and 30 on the right eye. Five patients underwent surgery on both eyes during a single surgery. In seven patients repeat surgery was necessary. One patient dropped out due to malignant genesis of the stenosis. The results of 56 patients counting 61 procedures overall were available at 6 months follow-up. Intubated and non-intubated groups were comparable in terms of age, sex ratio, side of operation, method of anesthesia and surgeons.

33 surgeries were performed with intubation of a silicone stent tube, 28 without intubation. Within the intubated group 27 of 33 procedures achieved anatomical success at six months follow-up (81.8%). Moreover, in 26 cases the procedure led to functional success (78.8%). In two patients, irrigation was possible, despite the lack of functional success and in one patient functional success was recorded though irrigation was not possible. In 25 cases neither anatomical nor functional failure was recorded (75.8%).

**Fig. 2:**
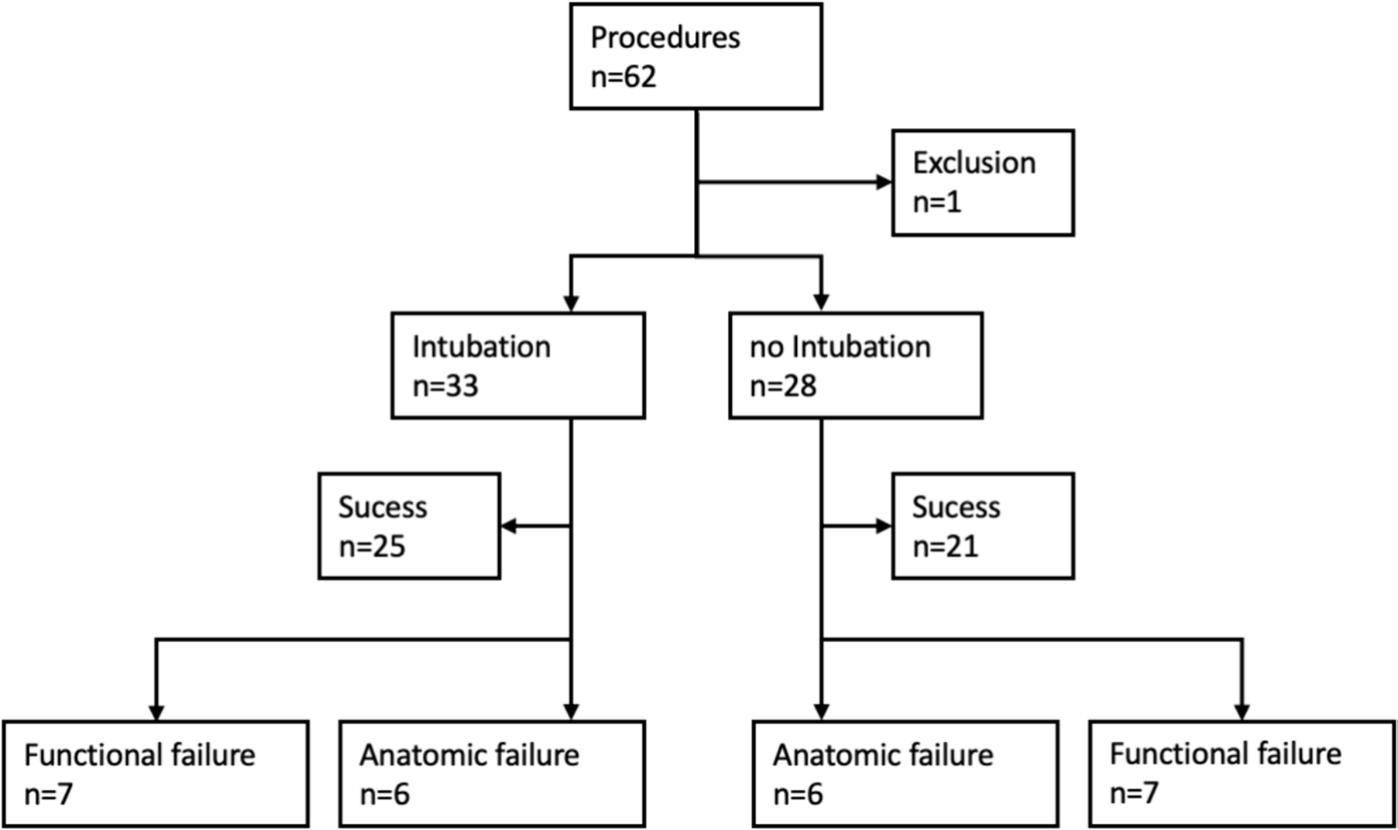
Outline of participant flow and group allocation after randomization

Among the 28 procedures of the non-intubated group 22 procedures achieved anatomical success at 6 months follow-up (78.6%). Functional success was recorded in 21 cases (75.0%). In one case irrigation was possible, though functional success was not achieved. In 21 cases neither anatomical nor functional failure was recorded (75.0%). The flow chart gives an overview of the total number of successful and failed procedures. The mean age represented in the failed cases was 66.27 years (SD = 21.46) (Fig. 2).

Table 1 shows the mean score for epiphora at each follow up. Patients without intubation showed lower mean scores for epiphora. Nevertheless, the level of significance was not reached (*p* > 0.10). Comparing the three follow-ups regarding changes in time and looking at patients with and without SSTs separately, no significant differences were found either (*p* > 0.28).

**Table 1:** Mean value representation of the clinical symptom epiphora in comparison between use/non-use of a silicone intubarium

		Mean	Standard deviation	N	*p* Value
Epiphora at 1st Follow up	With intubarium	1.61	1.202	31	.11
	Without intubarium	1.12	1.013	25	
	total	1.39	1.139	56	
Epiphora at 2nd Follow up	With intubarium	1.19	1.400	31	.59
	Without intubarium	1.00	1.225	25	
	Total	1.11	1.317	56	
Epiphora at 3rd Follow up	With intubarium	1.16	1.635	31	.85
	Without intubarium	1.08	1.498	25	
	Total	1.13	1.562	56	

Comparing the patency assessed by the irrigation of the nasolacrimal duct between patients with and without intubation no significant difference was found (*p* > 0.05).

Table 2 gives an overview with absolute numbers of patient’s patency with and without silicone stent at the second and third follow-up. Three patients refused irrigation. Despite there being a difference in the proportion of patency between the two groups, this difference remained insignificant after applying Wald’s test (*p* > 0.16).

**Table 2:** Patient’s patency with and without silicone stent at the second and third follow-up

		Success	Patients	Portion	*p* value
3 months Follow Up Patency = yes	With intubarium	26	31	0.839	.16
	Without intubarium	19	26	0.731	
6 months Follow Up Patency = yes	With intubarium	25	31	0.806	.47
	Without intubarium	20	25	0.800	

Patients were asked to rate the following side effects: encrustations of the nose and olfactory reduction. Neither appeared related to EN-DCR (rated by patients on a scale from 0 to 5, the mean score for encrustations was 0.3, for olfactory reduction 0.05).

The mean duration of all surgeries was 37.7 min. The mean time for surgeries with the insertion of a SST was 41.3 min. When no SST was used, the mean duration was 32.2 min. The mean duration of successful surgeries was 37.6 min, the mean duration of failed surgeries was not significantly longer with 37.9 min. Furthermore, no significant difference in duration could be found between failed cases with or without use of SST (37.5 min vs. 35.7 min).

In total, 10 different doctors performed the operations. All results were controlled for bias caused by surgeons. No distortions were found in this respect.

## Discussion

In the last decades minimal invasive procedures have become more and more popular. The endoscopic DCR has shown to have certain advantages compared to the external DCR, such as avoidance of a cutaneous incision and associated scar, less disruption of the medial canthal anatomy (including the deep pretarsal orbicularis muscle, the medial canthal tendon and various other structures), preservation of the nasolacrimal pump function, decrease in intraoperative hemorrhaging and postoperative infection as well as avoidance of any damage caused to peripheral fibers of the zygomatic and buccal branches of the facial nerve. [18–20] Therefore, endoscopic DCR has gained considerably in popularity [34–36].

The most common causes of failure in DCR surgery are inadequate osteotomy size or location, cicatricial closure of the ostium, failure in locating the sac and inadequate sac opening, obstructive septal deviation, synechia, granulation tissue and scarring. [7, 21] [22] Keren et al. showed a significantly higher rate of failure in patients with diabetes mellitus due to wound ulceration, impaired healing and chronicity. [23] However, other predictive factors with a higher risk of failure are discussed in the literature, such as young age, systemic inflammatory diseases as sarcoidosis or granulomatosis with polyangiitis and tumor diseases. [24–26] The surgeon’s experience seems to have an additional impact on the outcome of the surgery. Önerci et al. described a significantly higher success rate when experienced surgeons performed the operation. [7] Meanwhile, Ali et al. could not confirm this significant difference in success rates depending on the surgeon’s experience in their study on a similar subject. [27] This, however, is likely due to the progress achieved in the 14 years lying between these two studies, regarding higher quality endoscopic instruments and imaging systems, deeper anatomical understanding, as well as the advancement in the techniques of the surgery itself.

The benefits of a silicone stent remain controversially discussed. Chong et al. found no statistical difference in success rates in their prospective randomized trial comparing patients with and without intubation (96.3% vs. 95.3%). [28] Nor did Kang et al. or Orsolini et al. report any statistically significant differences in their meta-analysis comparing endoscopic DCR with and without SST intubation in regards to the surgical success rate and the postoperative complication rate. [29] Nevertheless, their respective conclusions differ. Orsolini et al. concluded in their meta-analysis that the use of SSTs slightly increases the chance of success of endoscopic DCR showing success rates of 94.0% with stent placement versus 90.6% without stent placement. Kang et al. concluded that the success and complication rate for endoscopic DCR is not influenced by the use of silicone intubation.

### Success rates

Back in 2003, Olver pointed out the importance of defining success, distinguishing between functional and anatomical success and a standardized follow-up procedure and time. [30] In many previous studies functional success was found to be lower than anatomical success. In the present study most cases of anatomical failure also reported functional failure. In one case of anatomic failure, functional success could nevertheless be achieved (patient with SST). Three patients, in which irrigation was possible, showed functional failure (two with SST, one without SST). After six months, the overall success rate in the intubated group was 75.8% (according to the literature the anatomical success rate with 81.8% was higher than the functional success rate with 78.8%). Among the non-intubated group, the overall success after six months was at 78.6% (with both anatomical and functional success at 75.0%). This difference did not reach levels of significance.

### Demographic and other factors

With a mean age of 66.27 years (SD = 21.46) in failed procedures, no significant difference to the mean age of the entire study population was found (mean age 68.65, SD = 17.65). In 20% of the failed cases patients were male, 80% were female. This does not diverge from this study’s collective gender ratio of 19.3% males and 80.7% females. The duration of the surgery was not found to have an impact on the surgical outcome either, with a difference of only 0.3 min (37.6 min mean duration of successful surgeries vs. 37.9 min mean duration of failed surgeries).

### Silicone stent tube

In this Study the use of a silicone stent tube did not affect the outcome of the surgery. Neither the incident nor the time of post-operative epiphora showed a statistically significant difference between patients with or without SST intubation. In this study, the measurement value of epiphora was collected based on the subjective patient sensation. However, no statistically significant difference between the two groups was found when using objective evaluation systems, such as irrigation of the nasolacrimal duct either. As Fayers et al. concluded in their study about functional and anatomical success, DCR surgery is mainly performed to benefit the patients’ comfort and improve quality of life, therefore, functional success should be prioritized over anatomical success. [31] As Ali et al. stated in their histological study the scarring phase of the tissues is usually complete by 3 months, however, subtle subepithelial changes continue to occur even up to 6 months. For this reason, a follow-up period of six months was set in the present study [32].

### Strengths and limitations

The strength of this study lies in its prospective, randomized design. In addition to this, the study had extremely few dropouts. One patient was excluded during the procedure due to malignant genesis of the nasolacrimal duct obstruction and three follow-up patients refused irrigation. Procedures were carried out by ten different surgeons. This reflects the reality of everyday life in university hospitals and other larger hospitals (results were controlled for potential bias caused by the surgeon. These results did not reach levels of significance. Looking forward, such a study could benefit greatly from a larger number of patients. Furthermore, a multi-center study with the goal of assessing a larger collection of data with a standardized surgical technique should be considered, given the fact that authors of meta-analyses on this subject have already brought up the limitations found in the analyses. [34–36] These can be traced back to just such differences in surgical techniques, lack of a standardized timepoint for stent removal, length of follow-up as well as different definitions of surgical success and postoperative treatment methods. In fact, there are several confounding factors that play an important role in the outcomes of a DCR.

Finally, pre-established scores to describe the subjective sensation of epiphora could help provide overall more consistent data. The more recently introduced FICI grading system by Ali et al. is a good start to standardize postoperative measurements and can help to improve the comparability of different studies. FICI is a simple and physician-friendly scoring and grading system to assess the health of a DCR ostium and makes it particularly easy to evaluate the postoperative healing progress [33].

## Conclusion

In summary, the success of DCR surgery is dependent on a complex conglomeration of several factors. There are few studies in the literature which focus singularly on the on the use of a silicone stent in DCR surgery. Most studies so far survey varying surgical techniques and patient factors. Different aspects of wound healing seem to have an important influence on the success rate of DCR surgery, e.g. consistent postoperative nasal care and specialist follow-up. In our view, on reflection of the available study results, the use of silicone stents is not the decisive and significant factor in achieving an anatomically and functionally successful outcome in (primary) DCR surgery.

## References

[1] Toti A. (1910) La dacriocistorhinostomia. Ann d’Ocul, CXIiii: 417

[2] Chandler PA (1936) Dacryocystorhinostomy. Trans Am Ophthalmol Soc 34:240–263 16693098 PMC1315579

[3] McDonogh M, Meiring JH (1989) Endoscopic transnasal dacryocystorhinostomy. J Laryngol Otol 103(6):585–587 2769026 10.1017/s0022215100109405

[4] Tsirbas A, Davis G, Wormald PJ (2004) mechanical endonasal dacryocystorhinostomy versus external dacryocystorhinostomy. Ophthal Plast Reconstr Surg 20(1):50–56 14752311 10.1097/01.IOP.0000103006.49679.23

[5] Watkins LM, Janfaza P, Rubin PAD (2003) The evolution of endonasal dacryocystorhinostomy. Surv Ophthalmol 48(1):73–84 12559328 10.1016/s0039-6257(02)00397-1

[6] Horn IS, Tittmann M, Fischer M, Otto M, Dietz A, Mozet C (2014) Endonasal nasolacrimal duct surgery: a comparative study of two techniques. Eur Arch Otorhinolaryngol 271(7):1923–1931 24190758 10.1007/s00405-013-2774-8

[7] Önerci M, Orhan M, Öğ O (2000) Long-term results and reasons for failure of intranasal endoscopic dacryocystorhinostomy. Acta Otolaryngol 120(2):319–322 11603798 10.1080/000164800750001170

[8] Roithmann R, Burman T, Wormald PJ (2012) Endoscopic dacryocystorhinostomy. Braz J Otorhinolaryngol 78(6):113–121 23306578 10.5935/1808-8694.20120043 PMC9448940

[9] Goldberg RA (2004) Endonasal dacryocystorhinostomy: is it really less successful? Arch Ophthalmol 122(1):108 14718306 10.1001/archopht.122.1.108

[10] Graue G (1932) An Soc mex de oftal y oto-rino-laryng, (9): 114

[11] Patrinely JR, Anderson RL (1986) A review of lacrimal drainage surgery. Ophthal Plast Reconstr Surg 2(2):97–102 3154549 10.1097/00002341-198601050-00008

[12] Allen K, Berlin AJ (1989) Dacryocystorhinostomy failure: association with nasolacrimal silicone intubation. Ophthalmic Surg 20(7):486–489 2779952

[13] Weber R, Hochapfel F, Draf W (2000) Packing and stents in endonasal surgery. Rhinology 38(2):49–62 10953841

[14] Saeed BMN (2012) Endoscopic DCR without stents: clinical guidelines and procedure. Eur Arch Otorhinolaryngol 269(2):545–549 21822856 10.1007/s00405-011-1727-3

[15] Longari F, Dehgani Mobaraki P, Ricci AL, Lapenna R, Cagini C, Ricci G (2016) Endoscopic dacryocystorhinostomy with and without silicone intubation: 4 years retrospective study. Eur Arch Otorhinolaryngol 273(8):2079–2084 26732693 10.1007/s00405-015-3876-2

[16] Madge SN, Selva D (2009) Intubation in routine dacryocystorhinostomy: why we do what we do. Clin Experiment Ophthalmol 37(6):620–623 19702714 10.1111/j.1442-9071.2009.02094.x

[17] Tadke K, Lahane V, Lokhande P (2020) Ostium characteristics and its relevance in successful outcome following endoscopic dacryocystorhinostomy. Indian J Otolaryngol Head Neck Surg. 10.1007/s12070-020-01970-2 36452842 10.1007/s12070-020-01970-2 PMC9702263

[18] Huang J, Malek J, Chin D, Snidvongs K, Wilcsek G, Tumuluri K (2014) Systematic review and meta-analysis on outcomes for endoscopic versus external dacryocystorhinostomy. Orbit 33(2):81–90 24354575 10.3109/01676830.2013.842253

[19] Vagefi MR, Winn BJ, Lin CC, Sires BS, LauKaitis SJ, Anderson RL (2009) Facial Nerve Injury during External dacryocystorhinostomy. Ophthalmology 116(3):585–590 19091406 10.1016/j.ophtha.2008.09.050

[20] Lee MJ, Park J, Yang MK, Choi YJ, Kim N, Choung HK (2020) Long-term results of maintenance of lacrimal silicone stent in patients with functional epiphora after external dacryocystorhinostomy. Eye 34(4):669–674 31527764 10.1038/s41433-019-0572-2 PMC7093529

[21] Bohman E, Dafgård Kopp E (2021) One-week intubation in external dacryocystorhinostomy– a report on long-term outcome. Orbit 40(4):287–291 32567441 10.1080/01676830.2020.1778737

[22] Chin J, Lam V, Chan R, Li CL, Yeung L, Law A (2020) Comparative study of stenting and ostium packing in endoscopic dacryocystorhinostomy for primary acquired nasolacrimal duct obstruction. Sci Rep 10(1):46 31913338 10.1038/s41598-019-57019-0 PMC6949294

[23] Keren S, Abergel A, Manor A, Rosenblatt A, Koenigstein D, Leibovitch I (2020) Endoscopic dacryocystorhinostomy: reasons for failure. Eye 34(5):948–953 31595028 10.1038/s41433-019-0612-y PMC7182564

[24] Kashkouli MB, Parvaresh MM, Modarreszadeh M, Hashemi M, Beigi B (2003) Factors affecting the success of external dacryocystorhinostomy. Orbit 22(4):247–255 14685898 10.1076/orbi.22.4.247.17255

[25] Sodhi PK, Verma L, Ratan S (2004) Young age - a risk factor for failure of dacryocystorhinostomy. Orbit 23(4):237–239 15590525 10.1080/01676830490521693

[26] Baek JS, Lee S, Lee JH, Choi HS, Jang JW, Kim SJ (2016) Predictors of silicone tube intubation success in patients with lacrimal drainage system stenosis. Korean J Ophthalmol 30(3):157 27247514 10.3341/kjo.2016.30.3.157 PMC4878975

[27] Ali MJ, Psaltis AJ, Murphy J, Wormald PJ (2014) Outcomes in primary powered endoscopic dacryocystorhinostomy: comparison between experienced versus less experienced surgeons. Am J Rhinol Allergy 28(6):514–516 25514488 10.2500/ajra.2014.28.4096

[28] Chong KKL, Lai FHP, Ho M, Luk A, Wong BW, Young A (2013) Randomized trial on silicone intubation in endoscopic mechanical dacryocystorhinostomy (SEND) for primary nasolacrimal duct obstruction. Ophthalmology 120(10):2139–2145 23672972 10.1016/j.ophtha.2013.02.036

[29] Orsolini MJ, Schellini SA, Souza Meneguim RLF, Catâneo AJM (2020) Success of endoscopic dacryocystorhinostomy with or without stents: systematic review and meta-analysis. Orbit 39(4):258–265 31662017 10.1080/01676830.2019.1677726

[30] Olver JM (2003) The success rates for endonasal dacryocystorhinostomy. Br J Ophthalmol 87(11):1431–1431 14609858 10.1136/bjo.87.11.1431 PMC1771892

[31] Fayers T, Laverde T, Tay E, Olver JM (2009) Lacrimal surgery success after external dacryocystorhinostomy: functional and anatomical results using strict outcome criteria. Ophthal Plast Reconstr Surg 25(6):472–475 19935252 10.1097/IOP.0b013e3181b81e9f

[32] Ali MJ, Mishra DK, Baig F, Naik MN (2016) Histopathology, immunohistochemistry, and electron microscopic features of a dacryocystorhinostomy ostium cicatrix. Ophthal Plast Reconstr Surg 32(5):333–336 26517203 10.1097/IOP.0000000000000530

[33] Ali MJ, Gupta A, Lakshmi CS, Ali MH (2022) The FICI grading for a dacryocystorhinostomy ostium. Eur J Ophthalmol 32(1):129–133 33579174 10.1177/1120672121994747

[34] Vinciguerra A, Nonis A, Resti AG, Bussi M, Trimarchi M (2020) Impact of post-surgical therapies on endoscopic and external dacryocystorhinostomy: systematic review and meta-analysis. Am J Rhinol Allergy 34(6):846–856 32703027 10.1177/1945892420945218

[35] Vinciguerra A, Resti AG, Rampi A, Bussi M, Bandello F, Trimarchi M (2023) Endoscopic and external dacryocystorhinostomy: a therapeutic proposal for distal acquired lacrimal obstructions. Eur J Ophthalmol 33(3):1287–1293 36254409 10.1177/11206721221132746 PMC10152216

[36] Vinciguerra A, Nonis A, Resti AG, Barbieri D, Bussi M, Trimarchi M (2021) Influence of surgical techniques on endoscopic dacryocystorhinostomy: a systematic review and meta-analysis. Otolaryngol Head Neck Surg 165(1):14–22 33228432 10.1177/0194599820972677

